# Immobilization of Bacteriophages in Ex Tempore Hydrogel for the Treatment of Burn Wound Infection

**DOI:** 10.3390/gels9080625

**Published:** 2023-08-03

**Authors:** Vladimir V. Beschastnov, Marfa N. Egorikhina, Alexander A. Tulupov, Igor E. Pogodin, Natalia Yu. Orlinskaya, Veronica. V. Antoshina, Irina Yu. Shirokova, Maksim G. Ryabkov

**Affiliations:** University Clinic, Privolzhsky Research Medical University, Nizhny Novgorod 603155, Russia; vvb748@mail.ru (V.V.B.); egorihina.marfa@yandex.ru (M.N.E.); tulupov.a.a@yandex.ru (A.A.T.); pogigevg@yandex.ru (I.E.P.); orlinskaya@rambler.ru (N.Y.O.); veronika_sh@list.ru (V.V.A.); shirokova.i@yandex.ru (I.Y.S.)

**Keywords:** wound dressings, hydrogel, bacteriophages, wound infection, ESKAPE, burn wounds, split-thickness skin grafts

## Abstract

The resistance of bacteria to antibiotics is a major problem for anti-bacterial therapy. This problem may be solved by using bacteriophages—viruses that can attack and destroy bacteria, including antibiotic-resistant ones. In this article, the authors compared the efficacy of topical bacteriophage therapy and systemic antibiotic therapy in the treatment of wound infections caused by ESKAPE pathogens in patients with limited (less than 5% of the body surface) full-thickness burns. Patients in the study group (*n* = 30) were treated with PVA-based hydrogel dressings saturated ex tempore with a bacteriophage suspension characterized by its lytic activity against the bacteria colonizing the wound. Patients in the control group (*n* = 30) were treated using etiotropic systemic antibiotic therapy, and the wounds were covered with gauze bandages soaked in an aqueous solution of povidone-iodine. An assessment of the decrease in the level of bacterial contamination of the recipient wounds in both groups was conducted after 7 days, and after that, free skin grafting was performed. On day 14 after free skin grafting, patients in both groups underwent incisional biopsy. The study group demonstrated an increase in the indices of proliferative activity (Ki-67), and angiogenesis (CD-31, VEGF) in the area of engraftment of the split-thickness skin grafts. The results indicate that PVA-based hydrogel wound dressings can be used as bacteriophage carriers for local antimicrobial therapy ahead of free skin grafting.

## 1. Introduction

Over 40% of burn wounds in patients undergoing inpatient treatment in burn centers are of full-thickness type [1,2,3] and require plastic closure. Relatively simple and safe, and thus one of the most widespread methods of plastic closure of burn wounds, is free skin grafting with a split-thickness skin graft [4]. However, subsequent lysis occurring with such split-thickness skin grafts is up to 30% [5,6], which leads to increases in the duration and cost of treatment due to the need for repeated plastic surgeries [7].

The major cause of such lysis of these grafts is infection of the burn wound [8,9]. The main pathogens responsible for this are those of the ESKAPE group: *E. faecium*, *S. aureus*, *K. pneumoniae*, *A. baumanii*, *P. aeruginosa*, and *Enterobactericeae* spp., as they are characterized by having acquired resistance to the medications used in antibacterial chemotherapy [10]. Irrational use of antibacterial chemotherapy medications, in addition to the consequent "selection" of resistance genes in hospital strains [11], has a cytotoxic and organotoxic effect that results in disruption of the reparative regeneration processes [12]. 

Taking into account the role of wound infection in the failed cases of free skin grafting, as well as the unintended consequences of antibacterial chemotherapy, there is a need to improve the methods of sanation of wounds colonized by antibiotic-resistant pathogens. The use of bacteriophages for such sanation of burn wounds is a promising method for preparing this type of wound for free skin grafting. 

It is important to note that the use of bacteriophages is limited and is not a universal remedy without unintended consequences. For instance, researchers and clinicians have concerns related to the fact that bidirectional interaction between bacteriophages and the patient’s immune status might lead to ineffectiveness of bacteriophage therapy or impaired immunological reactivity (allergic and autoimmune reactions) because of an interaction of immune system cells with bacterial viruses. However, the results of an experimental animal study conducted by authors from the USA and France headed by Roach D.R. with the goal to study the impact of the immune system on the effectiveness of bacteriophage therapy for pneumonia caused by *P. aeruginosa*, indicate that both bacteriophages and neutrophils, which are produced by the immune system of animals in response to contact with Pseudomonas aeruginosa lytic viruses, are required to treat inflammatory processes in lung tissue [13]. Based on the data received, the authors developed the immunophages synergy concept. Taking into account the multifactor interaction between the immune system and bacteriophages, one should remember that bacteriophages are not alien agents for the human body, as they permanently accompany the resident and transient representatives of its microbiome. Thus, the human immune system begins to adapt to bacteriophages from the first contact of the macro-organism with the bacterial cell. Moreover, the production of antibodies to bacteriophages is not equal to bacteriophage inactivation because inactivation largely depends on the antibody titer and specificity [14].

It should be noted that the ability of bacteriophages to self-replicate, the selectivity of effectiveness, and the ability to penetrate biofilms do not always guarantee clinical efficacy [15]. It is related to non-compliance with the basic principle of bacteriophages pharmacokinetics, which specified the need to create a concentration of bacteriophages in the area of clinical interest higher than the concentration of target bacteria by two orders of magnitude [16]. For instance, when wet-to-dry dressings are used as a “depot” for a bacteriophage solution, it evaporates and is diluted with the wound exudate, which results in a decrease in the initial concentration and lytic activity of bacteriophages in a few hours [17]. It is possible to maintain the concentration of bacteriophages in the wound at a level higher than the concentration of the target bacteria by immobilizing bacteriophages into carriers [18].

The most promising carriers which function as a matrix for bacteriophage immobilization are hydrogels [19]. Recently, hydrogels based on sodium alginate, hydroxypropyl methylcellulose, polyethylene glycol-4-maleimide, polyurethane, isopropylacrylamide-coallylamine, and hyaluronic acid methacrylate have been actively used to immobilize bacteriophages in the area of clinical interest [20]. In case of local bacteriophage therapy of wound infections, hydrogels based on polyvinyl alcohol (PVA) are some of the most promising carriers [21]. It should be emphasized that process capabilities related to the synthesis of PVA-based hydrogel on have not been depleted and are being actively improved. PVA hydrogels can be prepared by chemical or physical cross-linking. A detailed description of physicochemical properties of PVA hydrogels for both pharmaceutical and medical applications can be found in many excellent original papers and reviews [22,23]. Currently, the joint work of material researchers and clinicians is ongoing and the activities are focused on optimizing the synthesis technology for a hydrogel wound dressing that has the required characteristics for effective clinical use. For instance, using the microwave irradiation technology, researchers from Thailand developed a simple and economical technique for the synthesis of a stable hydrogel wound dressing with a high absorption capacity [24]. Due to regularly repeated hydroxyl groups in the PVA molecules structure, they are able to participate in development of hydrogen bonds, which ensure stabilization of immobilized bioactive substances and a prolonged therapeutic effect [25]. PVA-based hydrogels are highly absorbent, vapor-permeable, and inert to bacteriophages, whereas the bacteriophages solution is gradually released onto the wound surface without being replaced by wound exudate. The current data from a microbiological study of wound discharge show that the above-mentioned advantages of the PVA-based hydrogel wound dressings are supplemented by the possibility of saturation with bacteriophage solutions ex tempore, which is a basic requirement of bacteriophage therapy.

The study objective was to assess the efficacy and safety of the topical application of bacteriophages in a hydrogel for the sanation of burn wounds colonized by antibiotic-resistant microflora at the stage of their preparation for free skin grafting with split-thickness skin grafts.

## 2. Results and Discussion

### 2.1. Results of Microbiological Examination of Wound Discharge

The authors conducted an analysis of 300 results of microbiological studies of wound discharges sampled from 60 patients included in the study. In total, 378 bacterial strains were isolated, 291 (77%) of which were resistant to antibacterial medications. 

During the initial surgical treatment, the authors found that in 47 (78%) patients, the wound surface was colonized by a monoculture, and in 13 (22%) patients, by an association of two cultures from the resident microflora. The degree of bacterial contamination of burn wounds on day 1 of inpatient treatment was 1 × 10^3^ [1 × 10^2^; 1 × 10^4^] CFU/mL. The microorganisms identified during the primary microbiological study did not show multi-resistance to antibacterial medications.

Between days 7 and 10 of inpatient treatment, all patients demonstrated a change in the species composition of the wound microflora. In particular, the following strains were isolated most often in the form of mono-(88%) and mixed-infection (12%): *P. aeruginosa*—31 (46%), *K. pneumoniae*—18 (27%), and *S. aureus*—10 (15%). From the total of 67 strains of microorganisms isolated, 59 (88%) were MDR (multi-medication-resistant), and in eight (12%), the strains of *P. aeruginosa* were XDR (extensively medication-resistant), sensitive only to Colistin and Tiepenem. During this period, the median bacterial contamination of the burn wounds was 1 × 10^6^ [1 × 10^5^; 1 × 10^8^] CFU/mL.

After division of the patients into the study and control groups and appropriate antibiotic therapy had been conducted for 4 days, there was a decrease in the level of bacterial contamination of the burn wounds, with antibiotic-resistant microflora recorded in nine patients from the study group and six patients from the control group (*p* = 0.276). The bacterial contamination in both groups did not differ and was 1 × 10^5^ [1 × 10^4^; 1 × 10^6^] CFU/mL.

On the day of free skin grafting, corresponding to day 7 (D7) of local antibiotic therapy, a decrease in the degree of bacterial contamination (compared to D0) was detected in eleven patients of the study group and eight patients from the control group (*p* = 0.289). The median level of bacterial contamination of the burn wounds before free skin grafting in the groups had been 1 × 10^4^ [1 × 10^3^; 1 × 10^5^]. In connection with the local bacteriophage therapy, five patients of the study group achieved complete elimination of the target bacterium—*P. aeruginosa* (*p* = 0.0261). However, the vacated place did not remain “sterile”. In three cases, *P. aeruginosa* was replaced by *K. pneumoniae*, and in two cases, by cultures of potentially pathogenic intestinal microbiota from the *Enterobacteriaceae* spp. family: *Enterobacter cloacae* and *E. coli*, which were not polyresistant to antibiotics. 

During a comparative assessment of the results of the microbiological study of the burn wound discharges, in connection with antibacterial chemotherapy and the topical application of bacteriophages in hydrogel, the authors did not reveal any statistically significant differences in terms of the level of bacterial contamination. The data obtained indicate that the microbiological efficacy of topical application of lytic bacteriophages in hydrogel is equivalent to the microbiological efficacy of etiotropic antibacterial chemotherapy with systemic and local medications. 

On day 7 of local bacteriophage therapy, two patients in the study group demonstrated resistance of the *P. aeruginosa* colonizing their wound surfaces to polyvalent pyobacteriophages (scientific development and production center NPO “Microgen” JSC, Ufa). However, they were sensitive (with a high level of lytic activity) to the Pseudomonas aeruginosa bacteriophage (pyobacteriophage from the scientific development and production center NPO “Microgen”, Nizhny Novgorod, Russia). The authors’ clinical experience of using bacteriophages is consistent with published data that the development of resistance of the target bacteria to the bacteriophages in use is a natural and often inevitable outcome [26]. The tactic in these cases is regularly to change the officially approved series of bacteriophage suspensions used in the affected burns departments.

### 2.2. Results of Safety Assessment of Antimicrobial Therapy of Wound Infections

During the follow-up period, no allergic reactions, nor any development of local infectious processes were detected in the groups. 

### 2.3. Results of Histological Examination

The histological examination data, in general, confirmed successful engraftment of the split-thickness skin grafts in all patients. However, the histological structure of the split-thickness skin grafts and of the underlying tissues of the burn wounds of the patients in the study and control groups varied (Table 1). 

**Figure 1:**
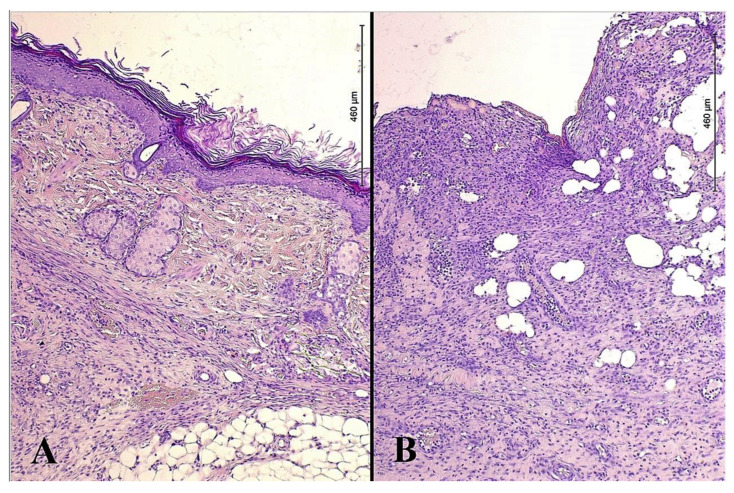
Microphotos of tissues of burn wounds closed with split-thickness skin grafts in patients of the study (**A**) and control groups (**B**); stained with hematoxylin and eosin; ×200.

The newly formed epithelium in between the perforation lumens of the split-thickness skin graft demonstrated extensive growth, with signs of the formation of skin derivatives. In the study group, there was a well-defined papillary layer under the epithelium base membrane; this layer was formed of loose connective tissue that protruded into the epithelium as papillae. This contrasted with the tissue samples from the control group, where a thin, even epithelial layer of low quality with pronounced leukocyte infiltration was found (Figure 1).

Quantitative analysis of morphometric indicators of reparative regeneration provided statistically significant differences for the groups in terms of the thickness of the newly formed epidermis (*p* = 0.036) and the proportion of newly formed capillaries (*p* = 0.011) in the lumen between the recipient wound and the split-thickness skin graft (Table 2).

### 2.4. Results of Immunohistochemical Study

**Figure 2:**
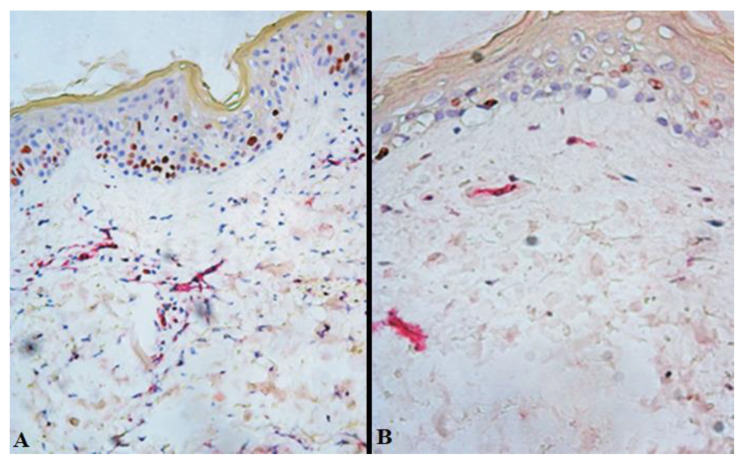
Expression of Ki-67 in the burn wound tissues closed with split-thickness skin grafts from patients of the study (**A**) and control (**B**) groups on day 7 after free skin grafting (D_14_). Brown staining—Ki-67-positive cells; red staining—membranes of endothelial cells; ×200.

On day 7 after free skin grafting, immunohistochemical staining of sections from the study group showed increased proliferation of the endothelial cells of the dermis compared with the control group (D_14_) (Figure 2, Table 2). For instance, the index of proliferative activity on day 7 after free skin grafting (D_14_) in the control group was 16 [13; 24] %, whereas in the study group it was 30 [22; 33] % (*p* = 0.017).

**Figure 3:**
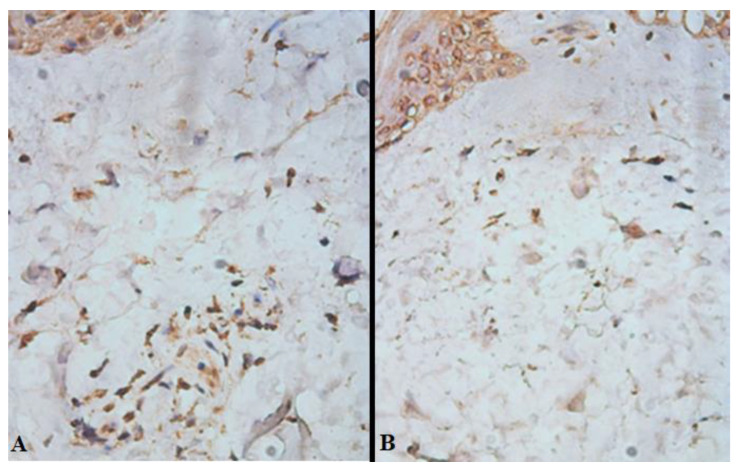
Expression of biomaterial to VEGF in the lumen between the recipient wound and the split-thickness skin graft of the study (**A**) and control (**B**) groups on day 7 after free skin grafting (D_14_). Brown staining—cells expressing receptors to VEGF; ×400.

On day 7 after free skin grafting (D_14_), transverse sections revealed that in the lumen between the recipient’s wound and the split-thickness skin graft, the proportion of cells expressing receptors to VEGF in such wounds of patients in the study group was 24 [21; 28] %, and in the control group—17 [14; 21] % (*p* = 0.032), while for receptors to CD31, it was 19 [16; 22] % in the study and 13 [11; 15] % in the control, respectively (*p* = 0.044) (Figure 3, Table 3).

The increase in proliferative activity and neoangiogenesis in the epidermis and upper layers of the dermis on day 7 after free skin grafting was probably due to the absence of any cytotoxic effects of the bacteriophages. The results obtained are of great clinical importance, as day 7 corresponds to the period of revascularization of the split-thickness skin graft and its integration into the tissues of the graft site, the efficacy of which determines the viability of the graft and the outcome of the free skin grafting in general.

### 2.5. Results of Free Skin Grafting with a Split-Thickness Skin Graft

The median of the engraftment area of split-thickness skin grafts in the control group was 91 [39; 98] %, while in the study group, it was 96 [76; 98] %, (*p* = 0.044). 

These differences in the indicator of engraftment area of the split-thickness skin grafts result from the fact that there was one case of subtotal lysis of the split-thickness skin graft in the control group. There were no such “extreme” values for the engraftment area of the split-thickness skin graft in the study group. 

Therefore, the results of the clinical study indicate that the local application of bacteriophages in a hydrogel at the stage of preparing a burn wound for free skin grafting is an effective and safe method of burn wound sanation. 

The advantage of a hydrogel created ex tempore is the possibility of using a bacteriophage that is relevant for the particular patient. The suggested method complies with the concept of personalized bacteriophage therapy [27], as it allows selecting lytically active bacteriophages based on the current clinical situation. This is important because in the case of a prolonged use of commercial wound dressings and medications containing bacteriophages from the same batch/series, as well as in the case of insufficient lytic activity of particular bacteriophages, the hospital microflora relatively quickly develops resistance to them [28].

The authors recorded a statistically significant decrease in proliferative activity, neoangiogenesis, and the development of newly formed low-quality epithelium in the studied tissues sampled from patients in the control group. This is probably related to the cytotoxic effects of the selected antimicrobial chemotherapy medications on the cellular components of the granulation tissue. The results obtained are consistent with the data of both experimental and clinical studies that confirm the cytotoxicity of the majority of local antiseptics; this cytotoxicity manifesting itself as a decrease in the growth of fibroblast cells and as destruction of microvasculature elements [29,30,31,32]. 

## 3. Conclusions

The described clinical experience indicates the efficacy and safety of the topical ap-plication of bacteriophages in a hydrogel for the sanation of burn wounds colonized by antibiotic-resistant microflora. During a comparative assessment of the results of the microbiological study of the burn wound discharges, in connection with antibacterial chemotherapy and the topical application of bacteriophages in hydrogel, the authors did not reveal any statistically significant differences in terms of the level of bacterial contamination (*p* = 0.289). The median level of bacterial contamination of the burn wounds in the groups had been 1 × 104 [1 × 103; 1 × 105]. On day 14 after using bacteriophages and free skin grafting, the study group demonstrated an increase in the indices of proliferative activity (Ki-67) and angiogenesis (CD-31, VEGF). This justifies the need for further research to provide a more detailed assessment of the clinical efficacy of this approach during surgical treatment of infected soft tissue wounds of other etiologies. The technique may then become the basis for the development of new antimicrobial therapy regimens to overcome the antibiotic resistance of infectious organisms associated with medical care.

## 4. Materials and Methods

In order to assess the efficacy and safety of our technique of local bacteriophage therapy for the sanation of burn wounds in the process of their preparation for free skin grafting with a split-thickness skin graft, we conducted a prospective cohort intervention study in the (adult) burns treatment department of the University Clinic, Privolzhsky Research Medical University during the period of 2020 to 2022.

In total, 60 patients with III-degree burn wounds colonized with antibiotic-resistant microflora and wound areas of up to 5% of the body surface participated in the study. The study was approved by the Local Ethics Committee of the Federal State Budgetary Educational Institution of the Higher Education “Privolzhsky Research Medical University”—Minutes of Meeting No. 2 of 4 March 2020.

All patients or their legal representatives were provided with relevant and clear information about the study and the need to provide their informed consent before becoming involved in it. Written informed consent was also obtained from the patients to publish this paper. 

Criteria for inclusion in the study:

− voluntary informed consent of patients or their legal representatives to participate in the study;
− age between 18 and 60 years;
− total area of burn damage to the skin up to 15% of the body surface, of which III-degree burns—up to 5% of the body surface;
− wound healing process in the regeneration phase;
− result of bacteriological examination of the wound discharge, confirming colonization of burn wounds by antibiotic-resistant microflora;
− sensitivity of the isolated microflora to bacteriophage suspensions of minimum “+++”, which corresponds to a simplified interpretation of “S”–sensitive.

Criteria for exclusion from the study:

− non-compliance with the inclusion criteria;
− burn disease in the burn shock or burn toxemia stage;
− thermal and inhalation injury;
− decompensation of concomitant diseases and progression of their complications, multiple organ failure, and cases when the risk to the health and life of the patient is higher than the planned treatment outcome;
− systemic infectious complications of the wound infection;
− skin damage by acids, alkalis, electricity, ionizing radiation;
− burns of functionally active areas (face, neck, feet, hands);
− participation in any other clinical trial or clinical approbation within the last three months before the start of the study;
− refusal of the patient or his legal representative to participate in the study;
− development of complications associated with the treatment;
− death of the patient.

Of the patients who participated in the study, 32 (53%) were men and 28 (47%) were women. In the study group (*n* = 30), there were 14 men, 16 women; in the control group (*n* = 30) were 18 men and 12 women (*p* = 0.437). The median age in the analyzed groups was 38 years [20; 56] years. There was no statistically significant difference between the groups in this respect (*p* = 0.314). The median age in the analyzed groups was 38 [20; 56] years. The median of the total area of patients’ skin burns recorded on day 1 of inpatient treatment was 12% [1; 15] of the body surface. In the study and control groups, the median of the total area of burns differed insignificantly: 11 [1; 13] and 12% [1; 14] of the body surface, respectively (*p* = 0.834). The median extent of III-degree skin burn in relation to the total body surface area in both groups was 3% [1; 5] of the body surface; there was no statistically significant difference (*p* = 0.922). The main causes of these thermal burns in the groups were flames—31 (52%) and hot water—27 (45%). In two cases, the cause of the burn was contact with hot metal. There were no differences between the groups in terms of the frequency of burns from a flame (*p* = 0.120).

The hypothesis of the study was that local bacteriophage therapy of a wound infection, in contrast to antimicrobial chemotherapy, at the stage of wound preparation for free skin grafting, contributes to an increase in the area of engraftment of a split-thickness skin graft due to the absence of cytotoxic impact on granulation tissue. 

The objects of the study were III-degree burn wounds colonized by antibiotic-resistant microflora, while the subjects of the study were the dynamic changes in the burn wound microflora, the area of engraftment of the split auto-dermal graft, the quality of the newly formed epithelium, and the characteristics of the reparative regeneration processes in the tissues of the wound surface, closed by the split auto-dermal graft. 

The criteria for setting the diagnosis included data obtained during examination of the skin burns, analysis of complaints, and history of the disease. Laboratory and instrumental diagnostic studies were conducted to determine the severity of each patient’s condition, to identify burn disease and its period, as well as any complications and concomitant diseases.

### 4.1. Systemic Use of Antibacterial Medications

In order to prevent infectious complications, all patients took systemic antibacterial medications. Before the results of microbiological studies of the wound discharges became available, antibacterial medications were prescribed empirically, taking into account the current epidemiological situation in the department. Etiotropic, systemic antibiotic therapy was prescribed, and, if necessary, adjusted, based on data regarding the antibiotic susceptibility pattern of the discharge of the burn wound. 

### 4.2. Primary Surgical Treatment 

Primary surgical treatment of the surface of each burn wound was conducted within 24 h from the time of patient admission to the hospital. The wounds and surrounding skin were lavaged with Prontosan solution (B. Braun, Melsungen, Germany). After that, the wounds were covered with sterile wet-to-dry gauze dressings with an aqueous solution of povidone-iodine (Haemofarm, Serbia).

### 4.3. Wound Discharge Sampling and Microbiological Examination

Wound discharges were sampled, both during primary surgical treatment and at subsequent dressing changes using a sterile cotton swab, by applying circular rotational movements from the center to the periphery of the wound surface. The obtained material was placed in sterile, hermetically sealed containers and transported to the bacteriological laboratory of the University Clinic of the Federal State Budgetary Educational Institution of the Higher Education “Privolzhsky Research Medical University” of the Ministry of Health of the Russian Federation to determine the species and quantitative composition of the wound microflora and its sensitivity to antibacterial medications and bacteriophages. A sample of the obtained biological material was applied to a sterile glass slide and stained according to Gram. 

The swab was examined under a microscope (Leica Microsystems Ltd., Wetzlar, Germany) and when microorganisms were found, their morphology and degree of seeding were recorded. The material was inoculated onto the surface of 5% blood agar according to the Drygalski modification of the sieving method and also transferred into sugar broth by dipping the swab with the biological material into it. The inoculated nutrient media were incubated at 37 °C for 24 h. After that, the dishes with cultures were examined and recorded for the presence or absence of growth on the 5% blood agar. In the case of an absence of growth on the dense nutrient medium, a sample from the sugar broth culture was inoculated onto 5% blood agar and incubated for 24 h. When bacterial growth was identified, colonies were seeded for species identification using a MALDI biotyper mass spectrometer (Bruker, MA, USA). When necessary, biochemical identification tests were conducted using the following identification kits: Staphy-test, Neferm-test, Entero-test, Encoccus-test, Strepto-test, and Neisseria-test (MIKROLATEST, Brno, Czech Republic) in accordance with the instructions for use and with subsequent automated recording of the identification results using the “Microbiological Monitoring System Microb-2” software (MedProject-3, Russia).

### 4.4. Local Conservative Impact on the Wound Surface

Assessment of the local conservative impact on the wound surface was conducted by considering the course of the phase of the wound healing process and the original degree of thermal damage to skin.

In the first phase of the wound healing process, dressings were changed at least twice a week with an assessment of the burn wound’s condition. An indication of the need for more frequent dressing changes was their excessive wetting with wound discharge. In the first phase of the wound healing process, the wounds were dry-treated using sterile wet-to-dry gauze dressings with an aqueous solution of povidone-iodine.

In the second phase of the wound healing process, for areas of mosaic lesions of the skin, the authors used Voscopran atraumatic bandages (“Novyje perevyazochnyje materialy” (New dressings) LLC, Moscow, Russia), as well as Argosulfan cream (Baush Health LLC, Moscow, Russia) to maintain a wet wound environment. 

During the transfer of some wounds to the third stage of the wound healing process, which was evidenced by epithelization of the Voscopran, atraumatic dressings with methyluracil as well as dressings with povidone-iodine solution were used to support the newly formed epithelium, in addition to the dressings with povidone-iodine solution allowing for dry-treating of the wound.

### 4.5. Differences in Local Treatment in Patient Groups

On the day of surgical necrectomy (D0), patients were randomly divided into the study and control groups. Systemic antibiotic therapy was stopped for patients of the study group (*n* = 30) and Polypran hydrogel dressings saturated with bacteriophage lytic suspension were applied to the surfaces of their wounds. Patients in the control group (*n* = 30) continued etiotropic systemic antibiotic therapy and the wounds were closed with sterile gauze dressings soaked in an aqueous solution of povidone-iodine. Dressings were changed once, three days before free skin grafting (D4).

### 4.6. Local Bacteriophage Treatment

**Figure 4:**
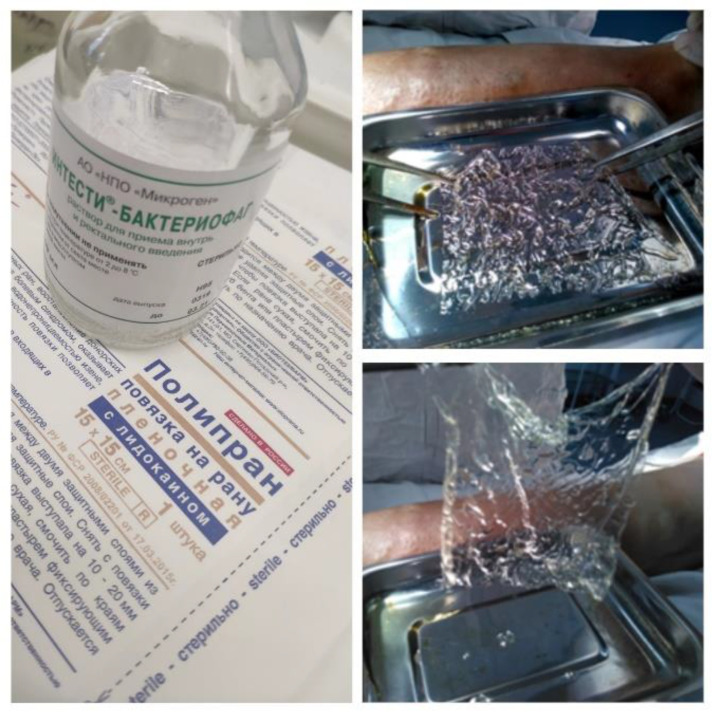
Saturation of the Polypran polymer film with bacteriophage suspension.

In the case of topical application of bacteriophages, the authors used Polypran polymer films saturated with bacteriophage suspension (NPO Microgen JSC, Russia) that had lytic activity against the pathogenic microflora colonizing the granulation surface of the burn wounds (Figure 4). In the case of topical application of bacteriophages, the authors used Polypran PVA-based hydrogels films saturated with bacteriophage suspension (Registration certificate No. FSR 2008/02201 dated 4 July 2022; TU 9393-010-52708501-2005, NPO Microgen JSC, Russia) that had lytic activity against the pathogenic microflora colonizing the granulation surface of the burn wounds (Figure 4). PVA-polymer was selected for this study because it is a hydrophilic bio-compatible polymer, is a suitable matrix for the immobilization of bioactive coatings, is non-toxic to humans, and its transparency allows evaluating the process of healing step-by-step [33]. 

The bacteriophage solution produced by the Federal State Unitary Enterprise “Scientific and Production Association for Immunological Preparations “Microgen” (Russia) is a sterile purified filtrate of bacterial phagolysates (with the Appelman activity of at least 10^5^). In addition to viral particles and their synthesis derivatives, the applied bacteriophage solutions contain a preservative 8-hydroxyquinoline sulfate or 8-hydroxyquinoline sulfate monohydrate equivalent to 8-hydroxyquinoline sulfate (0.0001 g/mL).

The authors suggested and have patented a technique for the topical application of bacteriophages in hydrogel. First, the microflora colonizing the graft site, and the sensitivity of these isolated pathogens to bacteriophages were identified. Then, a blank film was prepared for use by the addition of polyvinyl alcohol containing a phosphate buffer with a pH of 6.6–7.8 in the amount of (1–3) × 10^5^ mol/g. The blank film is a standard product—Polypran wound dressing (“Novyje perevyazochnyje materialy” (New dressings) LLC, Moscow, Russia). A total of 0.05 mL/cm^2^ of bacteriophage suspension were added to the prepared blank film, after which the film took on its gel form within 30 s. All Polypran samples were of the same area—225 cm^2^, and had absorbed the same volume (11.25 mL) of the suspension. 

**Figure 5:**
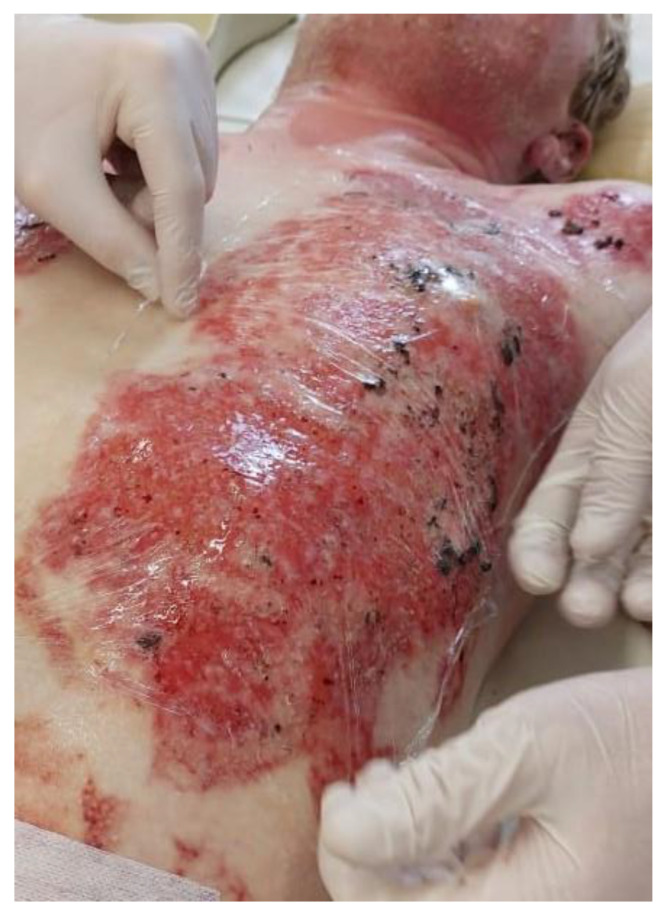
The burn wound is closed with a Polypran wound dressing saturated with bacteriophage lytic suspension.

After the Polypran had been saturated with the bacteriophage lytic suspension, the resulting hydrogel plate was modeled to the contour of the wound surface with no gaps or open areas (Figure 5).

The hydrogel plate was covered with a second dressing layer made of gauze soaked in saline. It was essential not to use antiseptics instead of saline as antiseptics have both anti-bacterial and antiviral activity and thus inactivate the bacteriophages. The next dressing was made after 4–5 days. 

Preservation of the bacteriophages’ lytic activity in the hydrogel was confirmed with both in vitro [34] and in vivo [35] studies; it was demonstrated that the bacteriophages’ lytic activity in hydrogels based on the Polypran wound dressing was maintained for 4–7 days. 

The authors considered the following microbiological criterion of efficacy of the suggested technique for local bacteriophage treatment: a decrease in the level of bacterial contamination of the recipient wound on day 7 (D_7_) of the local bacteriophage treatment.

### 4.7. Safety Assessment of the Suggested Technique for Local Bacteriophage Treatment of Wound Infection 

Safety assessment of the suggested technique was conducted during the entire period of its application. The authors recorded any indications of the occurrence and frequency of such undesirable consequences as allergic reactions or progression of the infectious process in the wound area. In line with the provisions of Article 23 of Federal Law No. 61 on the Circulation of Medicine dated 12 April 2010, the safety profile of the local bacteriophage treatment using the suggested technique was compared with the safety profile of the medical care standard.

### 4.8. Free Skin Grafting

Free skin grafting of the burn wounds was conducted with a split-thickness skin graft with a thickness of 0.3–0.4 mm. 

### 4.9. Assessment of the Free Skin Grafting Results

The result of free skin grafting was assessed by the area of engraftment of the split-thickness skin graft on day 14 after the grafting (D_21_). The engraftment area of the split-thickness skin graft was recorded using the LesionMeter mobile application (LesionMeter Team, USA) installed on a smartphone running the Android operating system [36]. The results of free skin grafting with a split-thickness skin graft were assessed using the *P* index, representing the proportion of the engraftment area resulting from the split-thickness skin graft, as calculated by the following formula:

$$P=\frac{S_{1}}{S_{2}}\times 100\%$$

where *S*_1_ is the area of split-thickness skin graft on the day of free skin grafting, cm^2^, and *S*_2_ is the area of split-thickness skin graft on day 14 after the operative procedure, cm^2^.

### 4.10. Incisional Biopsy and Storage of Biomaterial 

An incisional biopsy of the central part of the burn wound surface closed with a split-thickness skin graft was conducted using local anesthesia on day 7 (D_14_) and day 14 (D_21_) after free skin grafting. This was performed with a Derma Punch skin biopsy instrument (Medax, Italy) with a blade diameter and length of 3.5 and 6.5 mm.

The obtained column-like tissue fragment, 0.63 cm^3^ in volume, was placed in a sterile container with a fixation solution—10% neutral formalin in phosphate buffer (pH 7.2). The ratio of biomaterial and fixation solution was 1:10. The resulting biomaterial was delivered to the pathomorphology laboratory of the University Clinic of the Federal State Budgetary Educational Institution of the Higher Education, Privolzhsky Research Medical University, for histological and immunohistochemical examination. 

### 4.11. Histological Examination 

The fixed tissue samples were subjected to standard histological processing using an Excelsior ES machine (Thermo Scientific, Waltham, MA, USA). After processing, paraffin blocks were made based on TissuePrep 2 paraffin (Fisher Scientific, Waltham, MA, USA). Embedding into the paraffin blocks was conducted using a HistoStar embedding center (Thermo Scientific, Waltham, MA, USA). Serial transverse sections with a step of 5 microns were obtained on a Leica SM 2000 R sledge microtome (Leica micro-systems Ltd., Leider Lane Buffalo Grove, IL, USA). These sections were used to make micropreparations, which were stained with hematoxylin and eosin using a Gemini AS staining machine (Thermo Scientific, Waltham, MA, USA). 

The micropreparations were examined using a Leica DM I 3000B biomedical inverted expert-class microscope (Leica Microsystems Ltd., Mannheim, Germany) with ×5, ×10, ×20, ×40, ×100 objectives and ×10 eyepiece. Photofixation of the micropreparations was conducted with a microscope equipped with a Nikon Ds-Fi1 digital camera (Nikon Instruments Inc, Melville, NY, USA) and Nis-Elements BR image processing software (Nikon Instruments Inc, Melville, NY, USA).

Quantification of the histological structures of the burn wounds closed with split-thickness skin grafts was conducted using microscopic morphometry. The micropreparations were analyzed to calculate the number of cellular elements of the fibroblastic series that could be detected in 10 fields of view at a magnification of ×600. The state of any newly formed microvasculature was assessed by determining the number of developed microcapillaries in the lumen between the recipient bed and the split-thickness skin graft in 10 fields of view of the microscope at a magnification of ×600. The numerical values of the results were expressed as the average percentage in 10 fields of view. 

The development of a structurally high-quality, newly formed epithelium was the histological criterion of efficacy for the suggested technique of local bacteriophage treatment. Controls were conducted on day 7 (D_14_) and day 14 (D_21_) after the free skin grafting. 

### 4.12. Immunohistochemical Examination

A detailed examination of the proliferative activity and cellular differentiation of each of the burn wound tissues closed with the split-thickness skin graft was conducted using immunohistochemical research techniques involving monoclonal antibodies synthesized by immune cells belonging to one clone and evolving by the division of a single precursor cell.

Immunohistochemical staining was conducted in line with the reagent manufacturer’s recommendations. The staining protocol included preliminary deparaffinization of the sections in three Xyol alterations, rehydration in descending concentration alcohol baths, and washing in distilled water. Antigens were unmasked in a Decloaking Chamber Plus device (Biocare Medical, Pacheco, CA, USA) for 20 min at a temperature of 98–99 °C using citrate buffer of pH 6.0. To avoid background staining, the preparations were incubated in an endogenous peroxidase block and then washed in Tris-buffered saline buffer solution (Cell Marque, Rocklin, CA, USA) of pH 7.6. Immunohistochemical identification of immunocompetent cells (macrophages and Langerhans cells) in the biomaterial was conducted by staining sections with immunoperoxidase. 

The immunohistochemical examination was performed using monoclonal antibodies specific for:

− Ki-67 (GeneTex Inc., Irvine, CA, USA)
− CD31 (PrimeBioMed, Moscow Russia)
− VEGF (Dako Inc., Glostrup, Denmark)

Counterstaining of the micropreparations was conducted using a Leica Bond-Max immunohistostainer (Leica biosystems Ltd., Richmond, IL, USA), unmasking—using a water bath thermostat WB-4MS (Biosan, Latvia), and the incubation of antibodies—in a dry-air thermostat, the "TS-1/80” (“Smolenskoye SKTB SPU” JSC, Smolensk, Russia). Sections with a step of 4 microns were made using a Leica SM 2000 R sledge microtome (Leica microsystems Ltd., Buffalo Grove, IL, USA). The micropreparations were studied using a Leica DM I 3000B microscope (Leica Microsystems Ltd., Wetzlar, Germany) with subsequent photofixation of the sections with a Nikon Ds-Fi1 digital camera. Scanning and counting of the labeled cells was made with Aperio ScanScope Console software (LEICA, Wetzlar, Germany). 

Analysis of the immunohistochemical staining results was conducted using a semi-quantitative method for assessment of the degree of marker expression: blue—unlabeled cells; yellow—weak labeling; orange—medium labeling; and red—intensive labeling. 

Assessment of proliferative activity was conducted by counting the number of Ki-67-positive cells in 10 fields of view at a magnification of ×400. The proliferation index (PI) was determined using the following formula:

$$PI=\frac{n}{N}\times 100\%$$

where *n* is the number of labeled nuclei and *N* is the total number of nuclei in the field of view of the microscope. 

The authors considered the following immunohistochemical criteria of efficacy of the suggested technique for local bacteriophage treatment: an increase in proliferative activity and cellular differentiation of the burn wound tissues closed with a split-thickness skin graft. Controls were conducted on day 7 (D_14_) and day 14 (D_21_) after the free skin grafting.

### 4.13. Statistical Processing

Statistical processing of the obtained data was conducted using Statistica 10.0 software (StatSoft Inc., St Tulsa, OK, USA). Assessment of the statistical significance of differences between the quantitative comparisons of the groups was performed using non-parametric methods. The Wilcoxon test was used to compare two dependent (interrelated) groups, while the Mann–Whitney criterion was used to compare two independent (non-related) groups. Confidence intervals for the relative indicators were estimated using the Wilson technique. The selected parameters specified below have the following notations: Me—median, Q_1_—upper quartile, Q_3_—lower quartile, *n*—the size of the analyzed subgroup, *p*—the value of the statistical significance of the differences. In the M ± s formula, *M* is the arithmetic mean, and *s* is the standard deviation. The critical significance level was accepted as 5% (*p* ≤ 0.05).

## Figures and Tables

**Table 1:** Histological structure of split-thickness skin grafts and underlying tissues of burn wounds on day 14 after free skin grafting.

Group	Split-Thickness Skin Graft	Underlying Tissues
Study	Microstructure is preserved; there are minor dystrophic changes in cells; a lot of filled capillaries	Blood filling of capillaries “+++”; infiltration is characterized by a predominance of mononuclear leukocytes; moderate swelling
Control	Microstructure is preserved, mitotic items in the basal layer; dystrophic changes in cells; a moderate proportion of filled capillaries	Blood filling of the capillaries “++”; pronounced infiltration is characterized by a predominance of segmented leukocytes; moderate swelling

**Table 2:** Morphometric indicators of reparative regeneration processes in the area of split-thickness skin graft integration.

Morphometric Indicator	Group		
	Study	Control	*p*
Epidermis thickness, µm	96.04 [72.35; 121.65]	82.39 [58.1; 110.07]	0.036 *
Relative density of collagen fibers, %	70.8 [66.5; 77.9]	63.5 [52.4; 74.6]	0.085
Proportion of newly formed microcapillaries, %	49.2 [46.1; 51.3]	41.4 [36.3; 44.7]	0.011 *

* differences in the values of morphometric indicators in the area of integration of split-thickness skin grafts are statistically significant between the groups according to the Mann–Whitney criterion.

**Table 3:** Average proportion (%) of immunohistochemically stained cells in ten fields of view on day 7 (D_14_) and day 14 (D_21_) after free skin grafting.

Monoclonal Antibodies	Group	Check Date	
		D_14_	D_21_
		Average Proportion of Cells (%)	
VEGF	Study	24 [21; 28]	32 [28; 37]
	Control	17 [14; 21]	28 [22; 34]
	*p*	0.032 *	0.521
CD31	Study	19 [16; 22]	21 [17; 24]
	Control	13 [11; 15]	23 [19; 26]
	*p*	0.044 *	0.716
Ki-67	Study	30 [22; 33]	27 [21; 31]
	Control	16 [13; 24]	24 [19; 27]
	*p*	0.017 *	0.874

* differences in the values of morphometric indicators in the area of integration of the split-thickness skin graft are statistically significant between the groups according to the Mann–Whitney criterion.

## Data Availability

No new data were created or analyzed in this study. Data sharing is not applicable to this article.
